# Examining the factors associated with inpatients’ perception of overtreatment in Korea: a cross-sectional study

**DOI:** 10.1186/s12913-023-09563-9

**Published:** 2023-06-14

**Authors:** Jin Su Jang, Hyun Woo Jung

**Affiliations:** 1 Human Behavior & Genetic Institute, Associate Research Center, Korea University, Seoul, Republic of Korea; 2 Department of Health Administration, Graduate School BK21 - Graduate Program of Developing Global Experts in Health Policy and Management, Yonsei University, Wonju, Korea; 3 Division of Health Administration, College of Software and Digital Healthcare Convergence, Yonsei University, Yeonsedae-gil 1, Heungeop-myeon, Wonju-si, 26493 Gangwon-do Republic of Korea

**Keywords:** Overtreatment, Inpatient, Patient perception of care, Republic of Korea

## Abstract

**Background:**

Patients’ perception of receiving overtreatment can cause distrust in medical services. Unlike outpatients, inpatients are highly likely to receive many medical services without fully understanding their medical situation. This information asymmetry could prompt inpatients to perceive treatment as excessive. This study tested the hypothesis that there are systematic patterns in inpatients’ perceptions of overtreatment.

**Methods:**

We examined determinant factors of inpatients’ perception of overtreatment in a cross-sectional design that used data from the 2017 Korean Health Panel (KHP), a nationally representative survey. For sensitivity analysis, the concept of overtreatment was analyzed by dividing it into a broad meaning (any overtreatment) and a narrow meaning (strict overtreatment). We performed chi-square for descriptive statistics, and multivariate logistic regression with sampling weights employing Andersen’s behavioral model.

**Results:**

There were 1,742 inpatients from the KHP data set that were included in the analysis. Among them, 347 (19.9%) reported any overtreatment and 77 (4.42%) reported strict overtreatment. Furthermore, we found that the inpatient’s perception of overtreatment was associated with gender, marital status, income level, chronic disease, subjective health status, health recovery, and general tertiary hospital.

**Conclusion:**

Medical institutions should understand factors that contribute to inpatients’ perception of overtreatment to mitigate patients’ complaints due to information asymmetry. Moreover, based on the result of this study, government agencies, such as the Health Insurance Review and Assessment Service, should create policy-based controls and evaluate overtreatment behavior of the medical providers and intervene in the miscommunication between patients and providers.

**Supplementary Information:**

The online version contains supplementary material available at 10.1186/s12913-023-09563-9.

## Key Points

- Overtreatment experience in inpatient hospital settings varies according to patient sociodemographic and economic factors.
- Health care providers are suspected of performing overtreatment based on the inpatient’s socioeconomic status.
- Patients with private health insurance are more likely to experience overtreatment from medical providers.
- A public corporation should be given authority to monitor overtreatment.

## Introduction

The patient-centered care paradigm initiated in the early 2000s and led by the Institute of Medicine in the United States [1], proposed that the healthcare delivery system should provide customized services based on patient needs and consider the patient as the center of interactions within the system [1, 2]. In this regard, many studies have examined patient satisfaction and perception of care as one of the methods to evaluate the quality of care [3–5]. These studies used similar measurements such as communication with nurses or doctors, responsiveness of hospital staff, and quietness of the hospital [3–5]. However, they paid little attention to patients’ perception of overtreatment in evaluating the quality of care. This is quite an important issue because if patients feel that they have received overtreatment, they may lose trust in medical services, which goes against the patient-centered care paradigm.

Overtreatment is a concept that represents a situation wherein the amount of care that occurs is excessive [6–12]. Most studies have only researched overtreatment from the perspective of medical providers, as they are the ones who provide the treatment. For example, Lyu et al. [13] enlisted all the medical doctors listed in the American Medical Association master file and asked for their subjective perception of overtreatment. The authors reported that approximately 20.6% of overall medical care was unnecessary, and the most cited reason for overtreatment was fear of malpractice (84.7%). In general, studies have primarily been either audit studies or ones that focused narrowly on specific clinical interventions [13–16]. However, there has been no study to date that has analyzed patients’ perception of overtreatment.

One might suggest that it is not appropriate to investigate overtreatment from the patients’ perspective because it is the doctors who carry out the medical services, including overtreatment. The critics may insist that the patient’s perception of overtreatment cannot be a perfect proxy for the objective occurrence of overtreatment due to the two types of errors [17, 18]. The type 1 error occurs when patients report feelings of overtreatment although there was no overtreatment, whereas type 2 error occurs when patients do not report overtreatment concerns even though overtreatment was actually performed. However, these errors can occur when measuring physicians’ perception of overtreatment as well. In other words, there could be a substantial difference between the optimal and efficient treatment the doctor envisions and the best actual treatment.

We suggest that there are two dimensions to measuring overtreatment. The first is whether overtreatment has actually occurred, and the second is people’s subjective perception of overtreatment. The former defines overtreatment as a case where treatment “excessively and unnecessarily exceeded” the optimal standards; however, it is challenging to determine the optimal standards and measure the unnecessary excess using current scientific methods. Consequently, people’s subjective perception might be a more effective method, which can be subdivided into the perceptions of two groups: medical providers and patients. Based on the patient-centered paradigm [1, 19], it is essential to establish a mutual understanding between the doctor and patient. If patients persist in perceiving treatment as excessive even after health care providers reduce what may be considered overtreatment, their efforts would be unappreciated. In addition, without understanding the patient’s perspective, physicians might erroneously curtail good services.

This study mainly focused on inpatients rather than all patients for four reasons. First, outpatient services usually include simple diagnosis, testing, and treatment, in which case patients are usually sufficiently educated with the proper treatment by physicians. Moreover, outpatient treatment is typically short, providing little room for overtreatment. Second, admission to a hospital can be a traumatic and frustrating for many individuals [4]. Third, inpatient care is associated with much higher costs than costs for outpatient treatment [4, 20]. Fourth, the Korea Health Panel (KHP), which we used for this study, collected overtreatment data only for inpatients. Therefore, we set our strategy to examine factors associated with inpatient’s perception of overtreatment experiences in the Republic of Korea.

### Objectives

This study was the first to analyze factors associated with inpatients’ perception of overtreatment. Therefore, the study was exploratory in identifying what factors would be related to patients’ perceptions. Consequently, no specific variables were hypothesized to have an effect. However, we hypothesized that there would be systematic patterns in patients’ perception of overtreatment. In summary, this study examined associated factors affecting patients’ perception of overtreatment experience by applying medical provider-type factors to the Andersen healthcare utilization model.

## Methods

### Data source and study population

We used the 2017 KHP data from the National Health Insurance Services and Korea Institute for Health and Social Affairs. The KHP data were launched on 2008 and became popular for Korean researchers because of its publicly open data source and its many strengths in the healthcare sectors. One of the primary strengths is that it is representative of the population through its two-stage stratified random cluster sampling based on the Population and Housing Census. Once samples were selected, the KHP conducted a face-to-face survey with trained interviewers. The problem of information loss and recall bias errors was addressed by comparing self-reported data and receipt checks at the National Health Insurance Services. The original total number of individuals who participated in the 2017 KHP was 17,008, among whom 1,797 had been hospitalized at least once. After excluding 55 individuals with missing or inappropriate values for the variables, we analyzed data from 1,742 individuals.

Due to the nature of inpatients, the sample composition varies considerably by year; hence, we performed a cross-sectional study instead of a panel analysis. Since it could not be nationally representative, we weighted the sample when conducting a multivariate logistic regression. The KHP provides sample weight variables, which were calculated to address unequal selection probabilities and non-response bias and to align the sample distribution with the population distribution via post-stratification. To obtain population-representative estimates and account for the complex survey design, we employed cross-sectional weight variables to estimate the logistic regression coefficient. This study was approved by the Institutional Review Board of Yonsei University (approval number: 1041849-202207-SB-123-01).

### Variables

The dependent variable was a binary variable indicating the experience of overtreatment. The KHP investigated the following question for inpatients: “Have you felt that you received unnecessary examination or overtreatment?” The answers were classified as very positive, positive, normal, negative, very negative, or unsure. “Unsure” refers to the patient being not confident whether overtreatment was carried out by medical providers. Consequently, we excluded 19 “unsure” cases. The scale was diligently interpreted, especially in terms of “normal” responses. Generally, inpatient services are diverse, wherein the period of treatment is longer than that for outpatient services. “Very positive” responses indicate that the patient felt that most of the medical services he/she received were exceptionally excessive. “Positive” responses indicate that the patient felt that an excessive treatment existed at some point throughout receiving medical services. “Normal” responses indicate patients who sensed overtreatment only in certain areas of medical services. In other words, “normal” does not mean appropriate medical provision rather medical services that included minor overtreatment.

Because there were too few cases that provided detailed answers, we reorganized the answers as “yes” (1) or “no” (0). The very positive, positive, and normal responses were defined as having experienced any overtreatment, whereas the other responses were classified as not having experienced overtreatment (Table 1). Furthermore, we set the strict overtreatment variable for the sensitivity analysis, including normal as zero. Separating the variable of overtreatment as “any” and “strict” can be helpful in policy decision-making. For instance, any overtreatment can measure patients’ perception of overtreatment to a generous standard, while strict overtreatment can be a strict standard.

**Table 1:** Definitions of variables

Variable		Category	Code
Dependent variable		Any overtreatment	0: no (very negative and negative) 1: yes (very positive, positive, and normal)
		Strict overtreatment	0: no (very negative, negative, and normal) 1: yes (very positive and positive)
Independent variable	Predisposing factors	Gender	0: female, 1: male
		Age group	0: 18–29, 1: 30–39, 2: 40–49, 3: 50–64, 4: >65
		Educational level	0: college or higher 1: high school 2: middle school or less
		Region	0: capital city, 1: city, 2: rural
		Marital status	0: married, 1: single, 2: divorced, widowed, etc.
	Enabling factors	Economic activity	0: no; 1: yes
		Household income quintile	0: poor, 1: 2nd, 2: 3rd, 3: 4th, 4: rich
		Type of health coverage	0: NHI (employee), 1: NHI (employer), 2: Medical aid
		Status of PHI*	0: uninsured, 1: insured
	Need factors	Chronic disease	0: no, 1: yes
		Disabled	0: no, 1: yes
		Subjective health status	0: good, 1: mild, 2: bad
		Health recovery	0: no, 1: yes
	Medical service factors	General tertiary hospital	0: no, 1: yes
		General hospital	0: no, 1: yes
		Surgery	0: no, 1: yes

Note: NHI: National Health Insurance; PHI: Private Health Insurance; *: Primary factor

The independent variables were predisposing factors (gender, age group, educational level, region, and marital status), enabling factors (economic activity, household income, and type of health insurance), need factors (chronic diseases, disability, subjective health status, and health recovery) and medical provider-type factors (use of a general tertiary hospital, general hospital, and surgery) (Table 1). Among these variables, it needs more explanation for the health recovery. The KHP asked patients who had been hospitalized whether they think they have sufficiently recovered after discharge. Patients replied with either very uncomfortable, slightly uncomfortable, mostly recovered, completely recovered, ongoing hospitalization, or health checkup. This study regarded only “mostly recovered” and “completely recovered” as recovered cases. Death cases, health checkups, and ongoing hospitalized patients during the survey period were excluded because they were not on the scale of the level of recovery.

### Statistical analysis

First, the participants’ general characteristics concerning overtreatment were analyzed using the chi-square tests. Second, multivariate logistic regression analysis was performed to calculate the odds ratios (OR) and 95% confidence intervals (CI) of the effects of each independent variable. The Stata (version 15) statistical package program was used for all data analyses.

## Results

### General characteristics of participants with regards to overtreatment

Table 2 shows the participants’ general characteristics, representing the relationship between the patients’ perception of any overtreatment and the independent variables. A total of 347 (19.92%) inpatients felt undergoing any overtreatment. The study findings reveal that several predisposing, need, and medical service factors were associated with any overtreatment. Specifically, age group was significantly associated with any overtreatment, with patients aged over 40 years being more likely to perceive any overtreatment. Disabled patients indicated to relatively more overtreatment in hospitalized settings. Patients who rate their health as good were less likely to perceive any overtreatment compared to other patients. In addition, those who believed that their health had not recovered after hospitalization were more likely to report any overtreatment. Interestingly, the study indicates that the patients hospitalized in general tertiary hospitals were significantly more likely to report any overtreatment, unlike those in general hospitals. However, no clear relationship was found between enabling factors and any overtreatment.

**Table 2:** General characteristics of participants in relation to the experience of any overtreatment

Characteristic		Any overtreatment		Χ^2^ (P-value)
		No	Yes	
		n (%)	n (%)	
Gender	Male	520 (78.08)	146 (21.92)	2.709 (0.100)
	Female	875 (81.32)	201 (18.68)	
Age group (years)	18–29	136 (83.95)	26 (16.05)	9.589 (0.048)
	30–39	141 (85.45)	24 (14.55)	
	40–49	177 (74.37)	61 (25.63)	
	50–64	438 (80.51)	106 (19.49)	
	> 65	503 (79.46)	130 (20.54)	
Educational level	< Middle school	347 (80.89)	82 (19.11)	0.336 (0.845)
	High school	367 (80.31)	90 (19.69)	
	>College	681 (79.56)	175 (20.44)	
Region	City	532 (80.12)	132 (19.88)	0.001 (0.974)
	Rural	863 (80.06)	215 (19.94)	
Marital status	Married	1,036 (80.43)	252 (19.57)	0.558 (0.756)
	Single	102 (77.86)	29 (22.14)	
	Divorced, widowed, etc.	257 (79.57)	66 (20.43)	
Economic activity	No	673 (79.27)	176 (20.73)	0.682 (0.409)
	Yes	722 (80.85)	171 (19.15)	
Household income quintile	1 (lowest)	340 (81.15)	79 (18.85)	3.670 (0.452)
	2	315 (78.95)	84 (21.05)	
	3	244 (79.22)	64 (20.78)	
	4	230 (77.7)	66 (22.3)	
	5 (highest)	266 (83.13)	54 (16.88)	
Type of health coverage	NHI (employee)	946 (80.85)	224 (19.15)	4.492 (0.106)
	NHI (employer)	327 (76.76)	99 (23.24)	
	Medical Aid	122 (83.56)	24 (16.44)	
Status of PHI	Uninsured	197 (83.83)	38 (16.17)	2.394 (0.122)
	Insured	1,198 (79.5)	309 (20.5)	
General tertiary hospital	No	1,170 (81.36)	268 (18.64)	8.498 (0.004)
	Yes	225 (74.01)	79 (25.99)	
General hospital	No	930 (80.31)	228 (19.69)	0.115 (0.734)
	Yes	465 (79.62)	119 (20.38)	
Surgery	No	734 (81.19)	170 (18.81)	1.462 (0.226)
	Yes	661 (78.88)	177 (21.12)	
Chronic disease	No	887 (80.86)	210 (19.14)	1.119 (0.29)
	Yes	508 (78.76)	137 (21.24)	
Disabled	No	1,247 (80.76)	297 (19.24)	3.983 (0.046)
	Yes	148 (74.75)	50 (25.25)	
Subjective health status	Good	368 (84.99)	65 (15.01)	9.219 (0.01)
	Mild	599 (77.79)	171 (22.21)	
	Bad	428 (79.41)	111 (20.59)	
Health recovery	No	596 (75.06)	198 (24.94)	23.02 (0.000)
	Yes	799 (84.28)	149 (15.72)	
Total		1,395 (80.08)	347 (19.92)	1,742

Note: NHI: National Health Insurance; PHI: private health insurance

Table 3 presents the results of patients’ perception of strict overtreatment. A total of 77 (4.42%) inpatients felt undergoing strict overtreatment. Table 3 differs from the results of any overtreatment in Table 2 but are mostly similar. Although age group was associated with any overtreatment, whereas age was not significantly associated with strict overtreatment. In contrast, level of education was found to be associated with strict overtreatment. Consistent with the results of any overtreatment, enabling factors did not show any significant associations. In terms of need factors, subjective health status and health recovery showed similar results to any treatment, except for disability status and chronic diseases. Specifically, having a disability was not significant; however, chronic diseases were found to increase the patient’s perception of overtreatment. With regard to medical service factors, inpatients who received treatment at general tertiary hospitals and underwent surgical procedures were more likely to report strict overtreatment.

**Table 3:** General characteristics of participants in relation to the experience of strict overtreatment

Characteristic		Strict overtreatment		Χ^2^ (P-value)
		No	Yes	
		N (%)	N (%)	
Gender	Male	638 (95.8)	28 (4.2)	0.119 (0.73)
	Female	1,027 (95.45)	49 (4.55)	
Age group (years)	18–29	154 (95.06)	8 (4.94)	5.785 (0.216)
	30–39	162 (98.18)	3 (1.82)	
	40–49	229 (1.82)	9 (3.78)	
	50–64	523 (96.14)	21 (3.86)	
	> 65	597 (94.31)	36 (5.69)	
Educational level	< Middle school	410 (95.57)	19 (4.43)	8.245 (0.016)
	High school	447 (97.81)	10 (2.19)	
	>College	808 (94.39)	48 (5.61)	
Region	City	633 (95.33)	31 (4.67)	0.157 (0.692)
	Rural	1,032 (95.73)	46 (4.27)	
Marital status	Married	1,227 (95.26)	61 (4.74)	1.263 (0.532)
	Single	127 (96.95)	4 (3.05)	
	Divorced, widowed, etc.	311 (96.28)	12 (3.72)	
Economic activity	No	809 (95.29)	40 (4.71)	0.332 (0.564)
	Yes	856 (95.86)	37 (4.14)	
Household income quintile	1 (lowest)	403 (96.18)	16 (3.82)	5.442 (0.245)
	2	379 (94.99)	20 (5.01)	
	3	288 (93.51)	20 (6.49)	
	4	285 (96.28)	11 (3.72)	
	5 (highest)	310 (96.88)	10 (3.13)	
Type of health coverage	NHI (employee)	1,120 (95.73)	50 (4.27)	0.184 (0.912)
	NHI (employer)	406 (95.31)	20 (4.69)	
	Medical Aid	139 (95.21)	7 (4.79)	
Status of PHI	Uninsured	226 (96.17)	9 (3.83)	0.224 (0.636)
	Insured	1,439 (95.49)	68 (4.51)	
General tertiary hospital	No	1,383 (96.18)	55 (3.82)	6.915 (0.009)
	Yes	282 (92.76)	22 (7.24)	
General hospital	No	1,105 (95.42)	53 (4.58)	0.200 (0.654)
	Yes	560 (95.89)	24 (4.11)	
Surgery	No	873 (96.57)	31 (3.43)	4.368 (0.037)
	Yes	792 (94.51)	46 (5.49)	
Chronic disease	No	1,057 (96.35)	40 (3.65)	4.2 (0.04)
	Yes	608 (94.26)	37 (5.74)	
Disabled	No	1,473 (95.4)	71 (4.6)	1.021 (0.312)
	Yes	192 (96.97)	6 (3.03)	
Subjective health status	Good	426 (98.38)	7 (1.62)	10.82 (0.004)
	Mild	730 (94.81)	40 (5.19)	
	Bad	509 (94.43)	30 (5.57)	
Health recovery	No	742 (93.45)	52 (6.55)	15.65 (0.000)
	Yes	923 (97.36)	25 (2.64)	
Total		1,665 (95.58)	77 (4.42)	1,742

Note: NHI: National Health Insurance; PHI: Private Health Insurance

### Determinants of overtreatment experiences

This section reports the results of analysis on the factors associated with the inpatients’ perception of overtreatment using multivariate logistic regression in terms of weight. After weighting, the population size was 4,348,065. The effects of each independent variable on the inpatients’ overtreatment perception were estimated (Table 4).

**Table 4:** Factors associated with patients’ perception of overtreatment experiences

Characteristic		Any overtreatment			Strict overtreatment		
		OR	95% CI		OR	95% CI	
Gender (Male)	Female	1.311**	1.217	1.411	1.15**	1.008	1.311
Age group (18–29)	30–39	1.026	0.305	3.449	0.17	0.008	3.335
	40–49	1.863	0.467	7.436	0.243	0.003	18.04
	50–64	1.182	0.489	2.857	0.182	0.003	10.40
	> 65	1.085	0.457	2.576	0.179	0.001	25.00
Educational level (< middle school)	High school	0.954	0.651	1.398	0.561	0.056	5.583
	>College	1.031	0.838	1.266	1.563	0.116	20.92
Region (City)	Rural	1.192	0.000	21,638	1.041	0.000	15,339
Marital status (married)	Single	1.392**	1.026	1.891	1.221*	1.01	1.811
	Divorced etc.	0.986	0.72	1.35	0.715	0.303	1.686
Economic activity (No)	Yes	0.907	0.771	1.067	1.353	0.531	3.445
Households’ Income quintile (poor)	2	1.345*	0.965	1.874	1.732	0.522	5.745
	3	1.392**	1.239	1.564	2.462	0.373	16.21
	4	1.383*	0.965	1.980	1.874	0.260	13.49
	5(rich)	1.036	0.603	1.781	1.726**	1.518	1.963
Type of health coverage (NHI employee)	NHI employer	1.272**	1.062	1.523	1.186	0.512	2.744
	Medical Aid	0.781	0.368	1.655	1.039	0.327	3.293
Status of PHI (uninsured)	Insured	1.433*	0.941	2.182	1.388	0.372	5.175
General tertiary hospital (No)	Yes	1.73**	1.552	1.928	1.921*	0.809	4.559
General hospital (No)	Yes	1.381**	1.139	1.675	1.413	0.812	2.46
Surgery (No)	Yes	1.004	0.810	1.245	1.549**	1.236	1.941
Chronic disease (No)	Yes	1.308**	1.135	1.509	1.948**	1.258	3.017
Disabled (No)	Yes	1.378**	1.006	1.889	0.556	0.056	5.488
Subjective health status (Good)	Mild	1.626**	1.317	2.007	4.682**	3.244	6.759
	Bad	1.214	0.863	1.708	4.32**	3.291	5.672
Health recovery (No)	Yes	0.587**	0.395	0.871	0.635**	0.386	1.044
Constant		0.09	0.000	149.5	0.015*	0.000	4.653

Note: Any overtreatment classified the “normal” responses as positive (Yes: 1); Strict overtreatment classified the “normal” responses as negative (No: 0). NHI: National Health Insurance; PHI: private health insurance; CI: confidence interval. *P < 0.1, **P < 0.05

Overall, both any and strict overtreatment showed similar results. Regarding predisposing factors, females (OR of any overtreatment = 1.311; OR of strict overtreatment = 1.15) and non-married individuals (OR of any overtreatment = 1.392; OR of strict overtreatment = 1.221) were more likely to report experiencing overtreatment. Enabling factors showed similar relationships; however, there were somewhat different results between any and strict overtreatment. Higher-income groups were more likely to report both any and strict overtreatment, but statistical significances differed. In any overtreatment, income quintiles 2, 3, and 4, but not 5, were statically significant. In contrast, only quintile 5 was significant in strict overtreatment. With regard to health insurance, the National Health Insurance (NHI) employer coverage (OR of any overtreatment = 1.272; OR of strict overtreatment = 1.186) and private health insurance (PHI) coverage (OR of any overtreatment = 1.433; OR of strict overtreatment = 1.388) were significantly higher in any overtreatment but not for strict overtreatment. However, the ORs of strict overtreatment were similarly high.

For needs factors, the results indicated the inpatients with chronic diseases were more likely to report both overtreatments (OR of any overtreatment = 1.308; OR of strict overtreatment = 1.948). However, inpatients with disabilities showed different results between any and strict overtreatment. Patients with disabilities were more likely to report any overtreatment (OR 1.378, 95% CI [1.006–1.889]) but less strict overtreatment (OR 0.556, 95% CI [0.056–5.488]). The inpatients who self-reported their health status as not good were more likely to report experiencing both types of overtreatment. Furthermore, the participants who reported their health as recovered after being hospitalized were less likely to report experiencing overtreatment (OR of any overtreatment = 0.587; OR of strict overtreatment = 0.635).

Lastly, the medical services factors showed similar results between any and strict overtreatment. The patients admitted to the general tertiary hospital were more likely to report overtreatment (OR of any overtreatment = 1.73; OR of strict overtreatment = 1.921). Furthermore, patients hospitalized in general hospitals were more likely to report experiencing any overtreatment but there was no significant difference for strict overtreatment (OR of any overtreatment = 1.381; OR of strict overtreatment = 1.413). Lastly, patients who had received surgery were more likely to report having experienced strict overtreatment, but there was no significant difference for any overtreatment (OR of any overtreatment = 1.004; OR of strict overtreatment = 1.549).

## Discussions

This study was the first to examine factors associated with inpatient’s perception of overtreatment using Korean representative secondary healthcare data. Analyzing patients’ perception of having experienced overtreatment while being hospitalized is worthwhile, because such information contributes to the evaluation the quality of medical services and faithful implementation of a patient-centered treatment paradigm [1, 2, 19]. In addition, the estimated prevalence of overtreatment from patients’ perceptions can be used as basis for reasonably estimating the objective amount of overtreatment. Interestingly, this study found that 19.9% of inpatients perceived overtreatment in Korea (under the definition of any overtreatment), which is consistent with 20.6% reported by Lyu et al. [13] in their sample of American physicians. This is a remarkable coincidence in that the prevalence of overtreatment was perceived at a similar level despite these studies being conducted in different countries with different sample populations.

The determinants of patients’ perception of overtreatment identified in this study are as follows. First, in the results of predisposing factors, females and individuals with a single-marital status were more likely to perceive both any and strict overtreatment. Although not about overtreatment, this result is somewhat inconsistent with the findings of a meta-analysis that concluded there were no gender differences in satisfaction with regard to health care [21]. Similarly, Weisman et al. [22] did not find a pattern of gender differences in satisfaction; however, men benefited from the care more quickly, had better doctor-patient communication, and found the office staff courteous and helpful; whereas women were only more satisfied with customer service. In other words, the researchers concluded that gender differences in patient satisfaction depended on the type of medical service. Given our study showed that women were more likely to feel they are receiving overtreatment, medical personnel should actively focus on enhancing communication with female patients to lessen the perception of overtreatment.

Meanwhile, single individuals are more likely to perceive overtreatment than those who are married. This finding is interesting because the marital status can affect the communication between patients and doctors. Commnunication is regared as a major factor in reducing unnecessary examinations and treatements, positively affecting the overall medical process and treatment results [19, 23–25]. Patients may find it difficult to understand medical terminology and may not pay careful attention during a conversation with a doctor due to pain and symptoms. Thus, single patients may feel that communication has not been done properly. However, in the case of a married couple, communication can be facilitated because both the patient and spouse communicate with the physician. In fact, these interpretations seem to rely on the caregiver role rather than marital status.

The valuable findings of this study are that all enabling factors were statistically significant. The higher income group, employers enrolled in NHI, and people with PHI were more likely to experience overtreatment. The fact that higher income groups are more likely to report overtreatment could be due to the fact that they concern about the high opportunity cost of spending time on treatment, compared with lower income groups. It should be noted that the highest income group was significant only for strict overtreatment. We carefully infer that the wealthiest people do not suspect excessive treatment when they perceive the “normal” level of overtreatment because they have less concerns about the cost.

Additionally, it is worth noting that in the typical medical service setting, providers cannot officially obtain a patient’s financial information, only the type of health coverage. The Korean NHI system separates subscription qualification into three groups: employees, employers, and medical aid recipients [26]. Among them, there is no difference in NHI benefits for employees and employers, except in calculating premiums. Medical aid is a co-payment system funded by a national tax, which allows the economically poor or patients with high medical needs to use medical services at meager prices [26]. It is remarkable that people with PHI reported overtreatment, because patients who are privately insured are entitled to (partial) reimbursement of their medical bills. This might be due to a small trace of the provider’s moral hazard induced by PHI, which is thoroughly discussed in Korea [27, 28]. Lyu et al. [13] found that 70.8% of all physicians were more likely to perform unnecessary procedures when they expected more profits. Therefore, we raise suspicions that medical providers may be performing excessive treatment after inferring payment ability with regard to having PHI.

Meanwhile, all the need factors included in the model were significantly associated with the perception of overtreatment. Patients with chronic diseases and disabilities were more likely to report experiencing overtreatment. This may be due to the persistent and incurable nature of their condition, which can influence their thoughts and feelings about their treatment. In particular, an interesting finding is that disability was a significant factor for any overtreatment, but it was not significant for strict overtreatment. Rather, the disabled were less likely to perceive strict overtreatment. This may be because most individuals with disabilities receive overtreatment at a normal level rather than a positive or very positive level. Thus, determining if they are receiving excessive medical treatment would be difficult to differentiate because the amount of medical treatment they receive is inherently high.

We found that subjective health status was another factor that affected inpatients’ perception of overtreatment. Patients suffering mild or bad conditions were more likely to perceive overtreatment. However, since subjective health status was the state of health at the time of the investigation, there could be a time lag from the experience of hospitalization. In other words, the participant’s health condition at the time of the survey could be different than their health condition in the past when they experienced overtreatment. A variable to correct this is health recovery. This study confirmed that the inpatients who indicated that their health had recovered directly after being hospitalized perceived less overtreatment. In summary, the subjective health status variable should be regarded as a control variable in determining the factors of overtreatment. Alternatively, it can be argued that persistent care and monitoring services are crucial because the health status at the time of the survey affects the response of their past experience of overtreatment.

Other results showed that the patients hospitalized in the general tertiary and general hospitals are considerably more likely to report overtreatment. On the medical provider’s side, general (tertiary) hospitals would be motivated to expand revenue through overtreatment. Lyu et al. [13] showed that 20% of physicians exercised overtreatment due to pressure from the medical institution or management. On the contrary, patients may expect general hospitals to have a more outstanding quality of medical services than other medical institutions, such as clinics or health centers. Indeed, most patients with severe diseases are admitted to general tertiary hospitals [29]. Therefore, patients in tertiary hospitals may be more likely to report overtreatment because their severe condition calls for more reexamination, retreatment, and costs.

Another concern related to the type of hospital is the compensation model of hospitals. Lyu et al. [13] criticized the fee-for-service and flat salary physician compensation model driving overtreatment from physicians. According to fee-for-service, the more medical service provided, the higher the profit; thus, this encourages the medical provider’s overtreatment motivation [30]. In contrast, the flat salary model has no incentive for doctors to reduce unnecessary treatment and only makes them focus on avoiding malpractice claims [13]. The Korean national health system has employed fee-for-service mechanisms [31], constituting the main revenue source of hospitals. Moreover, hospitals have been adopting a salary compensation model for physicians. In other words, it can be inferred that Korea’s healthcare system has a compensation system that promotes overtreatment. Therefore, Korean health policymakers need to refer to other countries’ systems that provide financial incentives to hospitals to reduce the waste of medical resources, such as Maryland’s new global payment system [32].

Lastly, the patients who had received surgery were more likely to report strict overtreatment. Because surgery is mostly invasive, causes pain, and prolongs the length of hospital stay, it can come at a opportunity cost to patients, such as a loss of working income. Therefore, inpatients who received surgery reported more overtreatment. These findings suggest that a significant proportion of patients who underwent surgery perceive overtreatment to be beyond a positive level, while relatively few patients perceive it as a normal level. At the same time, it means that people who have not undergone surgery recognize the normal level of overtreatment considerably.

Patients who had not undergone surgery reported the “normal” level of overtreatment. In this regard, the advantage of sensitivity analysis is that it can assign different policy implications based on the interpretation of the normal level of overtreatment. The normal level has a low level of certainty; therefore, it is difficult to accept that overtreatment has actually occurred. However, because the number of patients experiencing normal overtreatment was higher than the number of patients experiencing excessive (positive) and exceptionally excessive (very positive) overtreatment, it can increase consumer power and cause collective complaints. Therefore, it is necessary to reinforce communication, emotional care, and monitoring activities after discharge from the hospital based on “any overtreatment.” In contrast, reports or perceptions of strict overtreatment can be used as a criterion to check whether there actually was overtreatment in medical services and what form it took. Korea has been operating the Health Insurance Review and Assessment Service (HIRA), which is a public corporation with jurisdiction and responsibilities for the quality of medical care services. HIRA reviews and evaluates medical providers’ bills, keeping an eye on appropriate patient treatment, overtreatment and overbilling, and the standard of medical care [33–35]. If HIRA uses this indicator, it will be able to narrow down the scope of overtreatment and overcharge investigations. Thus, HIRA could reduce the burden of extensive overtreatment investigations of hospitals and clinics across the country and effectively monitor overtreatment.

Based on the results of this study, we suggest empowering HIRA to intervene in the miscommunication between inpatients and medical providers. Furthermore, the authority of HIRA to monitor overtreatment and intervene where indicated at general tertiary hospitals should be strengthened.

### Limitation

Although this study estimated the prevalence of patients’ perception of overtreatment and the results are similar to that of a precedent study that surveyed physicians, the findings are not supported by prior studies with inpatients due to the lack of such studies. Accordingly, we were obliged to refer to the literature on quality of care and patient satisfaction when interpreting the results. In addition, a time gap bias might have existed because the time when the data was collected from participants differed; some patients may were surveyed during hospitalizing, whereas others were surveyed several months after discharge. Even though this study intended to mitigate problems by including a health recovery variable that reflects the hospitalization period, this sole variable may have been insufficient. Lastly, this study could not utilize the satisfaction with care (SWC) variable in the KHP data (Table 1 in the supplementary material). Patients who were dissatisfied with care may have considered the treatment they received to be unnecessary, leading to a perception of overtreatment. However, the relationship between SWC and perception of overtreatment could have a two-way causal relationship, because those patients who suspect overtreatment are likely to be dissatisfied with care. Therefore, we decided not to include the SWC variable.

## Conclusion

For decades, the overtreatment has only been investigated from the perspective of medical care providers. Although the health paradigm has shifted to patient-centeredness, patients have been excluded from the discussion of overtreatment. As such, careful attention should be paid to the perspective of patients in terms of lowering the perception of overtreatment. Hence, further studies on the extent of overtreatment perceived by patients are warranted.

## Electronic supplementary material

Below is the link to the electronic supplementary material.

Supplementary Material 1

## Data Availability

The KHP (https://www.khp.re.kr:444/eng/main.do) made the raw data we utilized available to the public with authorization. Access to the raw data is available by contacting the Korea Institute for Health and Social Affairs (KIHASA) and the National Health Insurance Services (NHIS). However, sharing processed data is prohibited according to their policy. Therefore, data will be available from the corresponding author upon reasonable request; the corresponding author will request permission from KIHASA and NHIS.

## References

[1] Sofaer S, Firminger K (2005). Patient perceptions of the quality of health services. Annu Rev Public Health.

[2] Baker A. Crossing the quality chasm: a new health system for the 21st century. British Medical Journal Publishing Group; 2001.

[3] Jha AK, Orav EJ, Zheng J, Epstein AM (2008). Patients’ perception of hospital care in the United States. N Engl J Med.

[4] Chumbler NR, Otani K, Desai SP, Herrmann PA, Kurz RS (2016). Hospitalized older adults’ patient satisfaction: inpatient care experiences. SAGE Open.

[5] Isaac T, Zaslavsky AM, Cleary PD, Landon BE (2010). The relationship between patients’ perception of care and measures of hospital quality and safety. Health Serv Res.

[6] Emanuel EJ, Fuchs VR (2008). The perfect storm of overutilization. JAMA.

[7] Bahadori F, Hakimi S, Heidarzade M (2013). The trend of caesarean delivery in the Islamic Republic of Iran. East Mediterr Health J.

[8] Daniels B, Dolinger A, Bedoya G, Rogo K, Goicoechea A, Coarasa J (2017). Use of standardised patients to assess quality of healthcare in Nairobi, Kenya: a pilot, cross-sectional study with international comparisons. BMJ Global Health.

[9] Elshaug AG, Watt AM, Mundy L, Willis CD (2012). Over 150 potentially low-value health care practices: an australian study. Med J Aust.

[10] Glasziou P, Straus S, Brownlee S, Trevena L, Dans L, Guyatt G (2017). Evidence for underuse of effective medical services around the world. The Lancet.

[11] King JJ, Powell-Jackson T, Makungu C, Hargreaves J, Goodman C (2021). How much healthcare is wasted? A cross-sectional study of outpatient overprovision in private-for-profit and faith-based health facilities in Tanzania. Health Policy Plann.

[12] Lagarde M, Blaauw D. Overtreatment and benevolent provider moral hazard: an experimental audit study in the south african private healthcare market. J Dev Econ. 2022:102917.

[13] Lyu H, Xu T, Brotman D, Mayer-Blackwell B, Cooper M, Daniel M (2017). Overtreatment in the United States. PLoS ONE.

[14] Hecker MT, Aron DC, Patel NP, Lehmann MK, Donskey CJ (2003). Unnecessary use of antimicrobials in hospitalized patients: current patterns of misuse with an emphasis on the antianaerobic spectrum of activity. Arch Intern Med.

[15] Lehnert BE, Bree RL (2010). Analysis of appropriateness of outpatient CT and MRI referred from primary care clinics at an academic medical center: how critical is the need for improved decision support?. J Am Coll Radiol.

[16] Chan PS, Patel MR, Klein LW, Krone RJ, Dehmer GJ, Kennedy K (2011). Appropriateness of percutaneous coronary intervention. JAMA.

[17] Hoffmann TC, Del Mar C (2015). Patients’ expectations of the benefits and harms of treatments, screening, and tests: a systematic review. JAMA Intern Med.

[18] Brownlee S, Chalkidou K, Doust J, Elshaug AG, Glasziou P, Heath I (2017). Evidence for overuse of medical services around the world. The Lancet.

[19] Park S, Kim H-K, Lee M (2023). An analytic hierarchy process analysis for reinforcing doctor-patient communication. BMC Prim Care.

[20] Quintana JM, González N, Bilbao A, Aizpuru F, Escobar A, Esteban C (2006). Predictors of patient satisfaction with hospital health care. BMC Health Serv Res.

[21] Hall JA, Dornan MC (1990). Patient sociodemographic characteristics as predictors of satisfaction with medical care: a meta-analysis. Soc Sci Med.

[22] Weisman CS, Henderson JT, Schifrin E, Romans M, Clancy CM (2001). Gender and patient satisfaction in managed care plans: analysis of the 1999 HEDIS/CAHPS 2.0 H adult survey. Women’s Health Issues.

[23] Tu J, Kang G, Zhong J, Cheng Y (2019). Outpatient communication patterns in a cancer hospital in China: a qualitative study of doctor–patient encounters. Health Expect.

[24] Epstein RM, Franks P, Fiscella K, Shields CG, Meldrum SC, Kravitz RL (2005). Measuring patient-centered communication in patient-physician consultations: theoretical and practical issues. Soc Sci Med.

[25] Sweeney L, Halpert A, Waranoff J (2007). Patient-centered management of complex patients can reduce costs without shortening life. Am J Manag Care.

[26] Choi J-W, Choi J-W, Kim J-H, Yoo K-B, Park E-C (2015). Association between chronic disease and catastrophic health expenditure in Korea. BMC Health Serv Res.

[27] You C-H, Kang S-W, Choi J-H, Kwon Y-D (2017). Effect of private health insurance on medical care utilization: six year unbalanced panel data model. Korean J Health Service Manage.

[28] Moon S-W, Kang TW, Oh H, Nam-Kyu S, So hyun J, Da-. J, et al. Basic analysis report on the 2017 Korean Health Panel Survey (1). National Health Insurance Service; 2019. Report No.: 2019-1-0005. in.

[29] Shin J, Ham D, Paik HY, Shin S, Joung H (2021). Gender differences in the risk of ischemic heart disease according to healthcare utiliation and medication adherence among newly treated korean hypertensive patients. Int J Environ Res Public Health.

[30] Jung CW, Park SC (2021). Fee-for-service health insurance and moral hazard of hospitals. Asia-Pacific J Risk Insurance.

[31] Kim D-J, Kim H-S, Oh M, Kim E-Y, Shin J-G (2017). Cost effectiveness of genotype-guided warfarin dosing in patients with mechanical heart valve replacement under the fee-for-service system. Appl Health Econ Health Policy.

[32] Oakes AH, Sen AP, Segal JB (2020). The impact of global budget payment reform on systemic overuse in Maryland.

[33] HIRA. Health Security System Wonju-si, Gangwon-do: Health Insurance Review & Assessment Service. ; 2022 [Accessed 2022 July, 1]. Available from: https://www.hira.or.kr/dummy.do?pgmid=HIRAJ010000006002

[34] Song SO, Jung CH, Song YD, Park C-Y, Kwon H-S, Cha BS (2014). Background and data configuration process of a nationwide population-based study using the Korean National Health Insurance System. Diabetes & Metabolism Journal.

[35] Park Y-T, Yoon J-S, Speedie SM, Yoon H, Lee J (2012). Health insurance claim review using information technologies. Healthc Inf Res.

