# Supplementary material for: Examining the factors associated with inpatients’ perception of overtreatment in Korea: a cross-sectional study
Source: BMC Health Serv Res. 2023 Jun 14;23:633. doi: 10.1186/s12913-023-09563-9 (PMC10268426; doi:10.1186/s12913-023-09563-9)
Supplement: Supplementary file 1 — Supplementary Material 1 [file 12913_2023_9563_MOESM1_ESM.docx]

- KHP asked participants, as “How satisfied were you with the overall service during your stay in the hospital?” and responses as: “Very Satisfied, Satisfied, Normal, Unsatisfied, Very Dissatisfied.” it allows caregivers to respond if the patient is not in a state to respond.
- We presented the results of the relationship between SWC and overtreatment in Table 1. In the results, a few cases answered that there was overtreatment very positively, even though the patient was very satisfied with the care. Also, some cases answered that there was overtreatment positively, even satisfied with care.

Table 1. The relationship between the satisfaction with care and the perception of overtreatment of inpatients.

|  | Overtreatment | | | | |
| --- | --- | --- | --- | --- | --- |
| Satisfaction with care | Very Positive | Positive | Normal | Negative | Very Negative |
| Very satisfied | 0 (0.00) | 2 (0.11) | 0 (0.00) | 45 (2.58) | 27 (1.55) |
| Satisfied | 1 (0.06) | 36 (2.07) | 133 (7.63) | 889 (51.03) | 81 (4.65) |
| Normal | 2 (0.11) | 23 (1.32) | 112 (6.43) | 289 (16.59) | 24 (1.38) |
| Unsatisfied | 2 (0.11) | 10 (0.57) | 25 (1.44) | 31 (1.78) | 4 (0.23) |
| Very unsatisfied | 0 (0.00) | 1 (0.06) | 0 (0.00) | 5 (0.29) | 0 (0.00) |
| Total | 5 (0.29) | 72 (4.13) | 270 (15.5) | 1,259 (72.27) | 136 (7.81) |
| Pearson Chi2 | 205.972 (P=0.000) | | | | |
